# Effects of thyroid hormone manipulation on pre-nuptial molt, luteinizing hormone and testicular growth in male white-crowned sparrows (*Zonotrichia leuchophrys gambelii*)

**DOI:** 10.1016/j.ygcen.2017.09.025

**Published:** 2018-01-01

**Authors:** Jonathan H. Pérez, Simone L. Meddle, John C. Wingfield, Marilyn Ramenofsky

**Affiliations:** a Department of Neurobiology, Physiology and Behavior, University of California, One Shields Avenue, Davis, CA 95616, USA; b The Roslin Institute, The Royal (Dick) School of Veterinary Studies, The University of Edinburgh, Easter Bush, Midlothian, EH25 9RG Scotland, UK

**Keywords:** Methimazole, Thyroid Hormone, Photoinduction, Gonadal Growth, Molt

## Abstract

Most seasonal species rely on the annual change in day length as the primary cue to appropriately time major spring events such as pre-nuptial molt and breeding. Thyroid hormones are thought to be involved in the regulation of both of these spring life history stages. Here we investigated the effects of chemical inhibition of thyroid hormone production using methimazole, subsequently coupled with either triiodothyronine (T3) or thyroxine (T4) replacement, on the photostimulation of pre-nuptial molt and breeding in Gambel’s white-crowned sparrows (*Zonotrichia leuchophrys gambelii*). Suppression of thyroid hormones completely prevented pre-nuptial molt, while both T3 and T4 treatment restored normal patterns of molt in thyroid hormone-suppressed birds. Testicular recrudescence was blocked by methimazole, and restored by T4 but not T3, in contrast to previous findings demonstrating central action of T3 in the photostimulation of breeding. Methimazole and replacement treatments elevated plasma luteinizing hormone levels compared to controls. These data are partially consistent with existing theories on the role of thyroid hormones in the photostimulation of breeding, while highlighting the possibility of additional feedback pathways. Thus we suggest that regulation of the hypothalamic pituitary gonad axis that controls breeding may be more complex than previously considered.

## Highlights

• Inhibition of thyroid hormone abolishes pre-nuptial molt and gonadal growth.
• Exogenous T4 or T3 restores pre-nuptial molt.
• Exogenous T4 but not T3 restores gonadal growth.
• Methimazole inhibition of thyroid production increases luteinizing hormone.

## 1 Introduction

Since, Rowan (1925) first identified that gonadal development in birds breeding at mid to high latitudes was triggered by increasing day length, photoperiodic regulation of avian behavior and breeding has been of continuing interest to researchers. Increasing spring photoperiod has been shown to initiate both pre-nuptial molt and to trigger gonadal development in birds (Dawson, 1999, Dawson, 2002, Dawson et al., 2001, King, 1968, Lesher and Kendeigh, 1941, Pandey and Bhardwaj, 2015). As pre-nuptial (also termed pre-alternate) molt is not observed in all species, control mechanisms remain obscure and it is often overlooked in studies of molt in favor of the more common and robust post-nuptial (also termed pre-basic) molt. However, the pre-nuptial molt contributes to the general vernal preparation for reproduction, as birds replace worn or winter plumage with a breeding plumage and for most species yearling birds molt into their adult plumage. As such, the pre-nuptial molt may have been evolutionarily conserved due to increased reproductive success as a result of plumage replacement, enhancing sexual ornamentation or breeding plumage that acts as an indicator of mate quality (Mulder and Magrath, 1994, Thompson and Leu, 1994 and references cited therein).

The integration of photoperiodic cues that trigger the changes in physiology, morphology, and behavior necessary to support the pre-nuptial molt and breeding life history stages occur through the neuroendocrine system. It has been suggested that photoinduction of vernal events (molt, migration, breeding) may utilize independent neural pathways (Moore et al., 1982). Experiments manipulating light intensity and wavelength have successfully demonstrated the separation of the breeding and vernal migratory photoinduction pathways (Wang et al., 2013), but there is no direct evidence to support or negate the independence of the pre-nuptial molt and breeding photoinduction pathways within the brain. To date the pathway by which photoperiodic information triggers the onset of pre-nuptial molt remains unknown (Wingfield and Silverin, 2002).

In contrast, the mechanisms by which increasing photoperiod leads to the expression of the breeding life history stage are now well documented (Kang and Kuenzel, 2015, Nakane and Yoshimura, 2010). Historically the coordination of reproduction has been attributed to regulation by the hypothalamic-pituitary-gonadal axis, specifically gonadotropin releasing hormone (GnRH-I), and the more recently discovered gonadotropin inhibitory hormone (GnIH) (Tsutsui et al., 2000). GnRH promotes the release of follicle stimulating (FSH) and luteinizing (LH) hormone from the anterior pituitary gonadotroph cells. LH and FSH bind to receptors in the gonads to promote primary sexual characteristics such as gonadal recrudescence, gametogenesis, and sex hormone production that in turn stimulate development of secondary characteristics, reproductive behavior and ultimately reproduction itself (Follett et al., 1974, King et al., 1966). GnIH has been shown to act on the hypothalamus, pituitary, and gonads to inhibit breeding in birds, probably by the inhibition of GnRH release and action (Bentley et al., 2009).

Recent work in Japanese quail (*Coturnix japonica*) has extended our understanding of these mechanisms linking the photoinduced release of GnRH to several candidate deep brain photoreceptors (DBP) within the avian brain. Neuropsin (Opn5) found in the paraventricular organ of birds (Nakane et al., 2010), vertebrate ancient opsin (VA-Opsin), which has been described in multiple hypothalamic regions (Halford et al., 2009), and melanopsin (Opn4) are all strong candidates for the DBP. Following photostimulation the DBP neurons then signal thyrotroph cells lining the pars tuberalis triggering the localized release of thyroid stimulating hormone (TSH) into the mediobasal hypothalamus. TSH stimulates the expression of deiodinase type 2 (DIO 2), responsible for the conversion of thyroxine (T4) to triiodothyronine (T3) (Ikegami et al., 2014, Nakao et al., 2008). The resulting increase in local T3 levels is thought to act on nearby glial cells associated with GnRH expressing neurons, leading to the exposure of GnRH nerve terminals, thus promoting the release of GnRH (Yamamura et al., 2006, Yoshimura, 2010).

Intriguingly, both the hypothalamic localization of DBP and the integral involvement of thyroid hormone (TH) signaling in breeding present strong parallels to the existing investigations of pre-nuptial molt. Lesion studies of the ventromedial hypothalamus (VMH) have been shown to successfully block the photoperiodically-induced expression of pre-nuptial molt and gonadal recrudescence in white-throated sparrows (*Zonotrichia albicollis*) (Kuenzel, 1974). These findings suggest the possibility of shared neural circuitry, by linking the perception of photoperiodic cues for both molt and breeding to the VMH. Similarly, TH have long been associated with molt and in particular the post-nuptial (Voitkevich, 1966). Studies of post-nuptial molt have shown that administration of exogenous TH will induce molt while thyroidectomy inhibits molt or replacement of artificially plucked feathers (Hahn et al., 1992, Kuenzel, 2003, Voitkevich, 1966). Yet, pre-nuptial molt remains understudied (Newton, 2009, Wingfield and Silverin, 2009).

To unravel the putative role of TH in breeding and pre-nuptial molt we used wild-caught wintering Gambel’s white-crowned sparrows (*Zonotrichia leucophrys gambelii*) held under naturally changing day length conditions. To date the majority of studies have used either artificially prolonged photoperiods to rapidly induce gonadal growth or have been conducted in predominately domesticated species also under artificial photoperiod regimes. Here we present our findings on the effects of chemical inhibition of TH production by methimazole and a subsequent TH replacement on pre-nuptial molt, LH secretion, and gonadal growth. Gonadal growth and circulating LH were included as measures of reproductive development to allow the disentangling of central effects within the hypothalamus from potential downstream actions. Based on the existing literature we predicted that inhibition of TH production would prevent both pre-nuptial molt, plasma LH increase, and gonadal growth. Furthermore, we tested the prediction that replacement therapy with either T3 or T4 would restore all three variables to control levels.

## 2 Materials and methods

### 2.1 Study species

The Gambel’s white-crowned sparrow is a photoperiodic, seasonally breeding songbird (Chilton et al., 1995) that displays a well-documented pattern of pre-nuptial molt and gonadal growth in captivity, as well as migrations and post-nuptial molt (Agatsuma and Ramenofsky, 2006, Farner et al., 1953, King et al., 1966). Exposure to natural or artificially increased photoperiods in captivity triggers the GnRH cascade with subsequent rapid elevation of LH plasma levels, and gonadal recrudescence (Farner et al., 1980, King et al., 1966, Wingfield and Farner, 1978). All animals reported here were part of a larger study on the role of thyroid hormones in vernal events including migratory behavior, which has been reported previously (Pérez et al., 2016). Monitoring of circulating thyroid hormones was conducted simultaneously throughout the larger experiment and initially published in our first report on the role of thyroid hormones in migratory behavior, but have been reproduced here for clarity and context.

### 2.2 Experiment 1: Inhibition of thyroid hormone using methimazole

Eleven male white-crowned sparrows were caught by a combination of walk-in traps and Japanese mist nets in the vicinity of the University of California Davis, Davis, CA, USA (N 38.554, W 121.738) in the late autumn of 2012. Sex determination was conducted in the field using minimum wing length of ≥75 mm to identify males (Fugle and Rothstein, 1985) and subsequently verified by laparotomy. Birds were housed in individual cages and locomotor activity monitored continuously by an infrared photodetection system Mini Mitter Acquisition System – Vital View (Sun River, OR) as previously described (Pérez et al., 2016). Birds were held on natural photoperiod (38° N) using light timers with an Astro feature that mimics natural changes in day length and given water and food (50:50 mixed seed and Mazuri Small Bird Maintenance Mini-Diet; PMI Nutrition International, LLC St. Louis MO) *ad libitum*. Birds were randomly assigned to control implant (n = 4) or methimazole (M8506; Sigma Aldritch, St. Louis MI) implant treatment groups (n = 7). Both control implant and treatment birds received two pre-sterilized 14 mm silastic implants (OD 1.96 mm, ID 1.477 mm), sealed with silicone sealant at both ends, inserted subcutaneously on the flanks beginning on February 21, 2013. The treatment implants were packed with methimazole powder, while control implants were empty. All methimazole implants were replaced every two weeks, controls underwent sham replacement surgery. Methimazole implanted birds also had methimazole in their drinking water (500 mg/L) to ensure sufficient dosage to achieve thyroid hormone suppression (Pérez unpublished data).

### 2.3 Experiment 2: Thyroid replacement

Thirty-two adult white crowned sparrows were caught as above in the vicinity of Davis, CA in January of 2014. Post mortem observations confirmed 29 males and 3 females were subjects in the study. As sex did not affect the results of the analyses for all variables examined, except testis length, females were included in all other analyses. Housing conditions were identical to those described in Experiment 1 above.

Birds were randomly assigned to four treatment groups: control, methimazole (M), methimazole + T3 (MT3), and methimazole + T4 (MT4). All birds except for the control group received methimazole via implants and orally, while controls received empty implants. Methimazole implants were renewed every two weeks. The methimazole + T3 (MT3) birds received a single 10 mm subcutaneous silastic implant packed with T3 (t2877; Sigma-Aldrich) while the methimazole + T4 birds (MT4) received a 12 mm silastic implant packed with T4 (t2376; Sigma-Aldrich). TH implants were replaced once over the course of the experiment to ensure continuous delivery of hormone.

All procedures for both experiments were conducted under UC Davis IACUC approval #17235, USFWS Permit MB813248, California State Permit SC-186 000519 and the number of birds used was minimized.

### 2.4 Molt

Body molt was scored every two weeks for three regions of the body: abdomen, back, and crown on a scale of 0 to 3 for intensity, adapted from Morton et al. (1969); flight feathers and most rectrices are not molted during the pre-nuptial molt and were thus not scored. A score of 0 was given when no molt was present, 1 given when up to ∼10% of the region was undergoing molt, 2 given for 10–50% of feathers, and a score of 3 for over 50% of feathers molting. Molt scores for the three regions were summed to provide a composite body molt score, referred to hereafter as molt score. Molt was scored in all birds at all time points by a single observer, blind to treatment group.

### 2.5 Laparotomies

Surgical laparotomy was used to determine testis size at the end of experiment 1. Birds were sedated with isofluorane gas provided through a vaporizer (2–4% with oxygen). Laparotomies were graciously performed by Professor Thomas Hahn (Univ. California, Davis) and testis length was measured to the nearest 0.1 mm using calipers.

### 2.6 Thyroxine assay

Thyroxine levels were measured via direct radioimmunoassay as reported previously (Pérez et al., 2016). Briefly, a standard curve was created by a nine fold serial dilution of a thyroxine standard from 50 ng/mL. Samples were run in duplicate using 20 μL of plasma for each duplicate. Water blanks, high and low standards were run to internally validate the assay. 20 μL of stripped white-crowned sparrow plasma was added to non-specific binding, total binding, standard curve and standard tubes. Rabbit polyclonal anti-thyroxine antibody diluted 1:500 was used as the primary antibody (GWB-7B9782 Genwaybio, San Diego CA). Following primary antibody addition, 250 μL of barbital buffer with bovine gamma globulins (15 mg/mL) and 8-Anilino-1-naphthalenesulfonic acid (3 mg/mL) was added to all tubes. The stock barbital buffer was prepared by dissolving 22.68 g of sodium 5,5-diethylbarbiturate (B0500; Sigma-Aldrich, St. Louis MO) and 0.65 mg sodium azide (8.22335 EMD Millipore; Sigma-Aldrich) in 1000 mL distilled water. Samples were then incubated for 90 min in a 37 °C water bath prior to the addition of goat anti-rabbit antibody (1:4 dilution; Antibodies, Inc. Davis CA). Samples were then incubated overnight at 4 °C and on the second day 250 μL of 5% polyethylene glycol was added to enhance pellet formation. Samples were centrifuged at ∼1910*g* for 30 min at 0 °C and the supernatant was aspirated from tubes and the radioactivity of pellets counted using a Cobra II Auto-Gamma Counter (Packard, Meriden CT). Average inter-assay variation was 13.6% and intra-assay variation was 6.98% with a detection limit of 8.44 pg of hormone per assay tube.

### 2.7 Luteinizing hormone assay

To measure plasma LH, we used the LH radioimmunoassay described previously (Sharp et al., 1987) with slight modifications. Briefly, the assay reaction volume was 60 μL, comprised of 20 μL of plasma sample or standard, 20 μL of primary rabbit LH antibody, and 20 μL of I125-labelled LH. The primary antibody was precipitated to separate free and bound I125 label using 20 μL of donkey anti-rabbit precipitating serum and 20 μL of normal rabbit serum. All samples were measured in duplicate in a single assay. The intra-assay coefficient of variation was 5% and the minimum detectable dose was set to 0.2 ng/mL. This radioimmunoassay has been used extensively to quantify plasma LH in many avian species (Ciccone et al., 2007, Fraley et al., 2013, Lal et al., 1990, Lea et al., 1991, Schaper et al., 2012), including multiple species of Emberizidae sparrows (Deviche et al., 2012a, Deviche et al., 2008, Deviche et al., 2012b, Meddle et al., 2002, Wingfield et al., 2012).

### 2.8 Statistical analyses

All statistical analyses were conducted in R (R Core Development Team, 2014). Circulating levels of T4 and LH were compared separately by linear mixed effects models in lme4 (Bates et al., 2014) and lmerTest (Kunznetsova et al., 2014) packages with fixed effects of Treatment, Day of Year, and the interaction of Day of Year and Treatment; the effect of repeated sampling was accounted for by the inclusion of bird id as a random effect. Testicular length was analyzed by Welsh’s *t*-test for Experiment 1 and by ANOVA for Experiment 2; between treatment differences were explored post hoc using Tukey’s HSD. Molt intensity was analyzed in the ordinal package using cumulative logistic mixed models with a logit link to account for the ordinal nature of the data (Christensen, 2015). Separate models were run for each experiment, but all models included fixed effects of treatment, day of year, and their interaction, as well as bird ID as a random effect to account for repeated sampling. Day of year was centered and scaled against itself to improve model convergence. All data are presented ± standard error of the mean (s.e.m.).

## 3 Results

### 3.1 Thyroxine levels

**Fig. 1:**
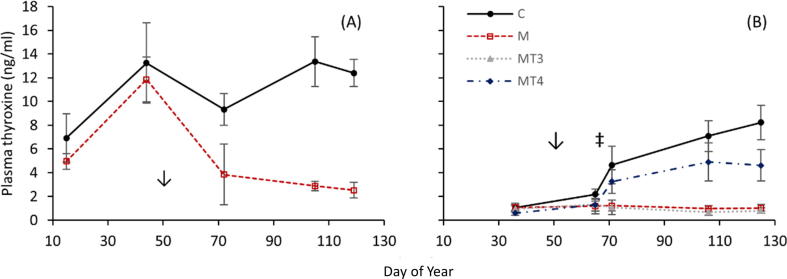
Plasma thyroxine levels as measured by radioimmunoassay in Gambel’s White-crowned sparrows following methimazole inhibition of thyroid hormone (A) and thyroid replacement (B). Treatments included control (C; A: n = 4, B: n = 7), methimazole (M; A: n = 7, B: n = 7), methimazole + T3 (MT3; B: n = 7) and methimazole + T4 (MT4; B: n = 8). Values are shown as mean ± s.e.m. Reprinted from Pérez et al. (2016), with permission from Elsevier.

As previously reported (Fig. 1; Pérez et al., 2016), methimazole treatment significantly reduced circulating levels of T4 in all birds. In the thyroid replacement experiment MT3 birds displayed thyroxine patterns matching those of Methimazole treated birds, while MT4 birds showed partial recovery of circulating thyroxine levels as compared to controls.

### 3.2 Gonadal growth

**Fig. 2:**
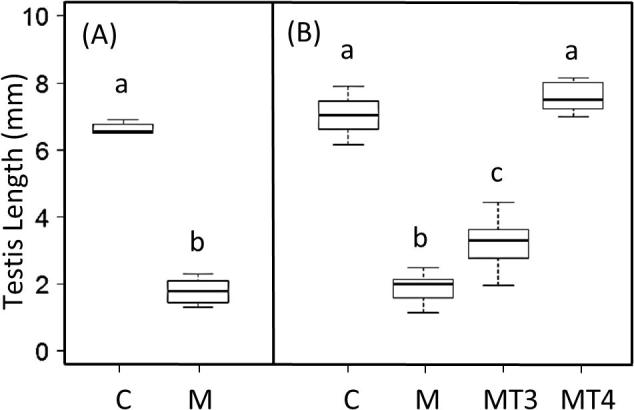
Testicular length for male Gambel’s White-crowned sparrows following methimazole inhibition of thyroid hormone (A) and thyroid replacement (B); experiments as measured at the end of the experimental period. Treatments included control (C; A: n = 4, B: n = 7), methimazole (M; A: n = 7, B: n = 7), methimazole + T3 (MT3; B: n = 7) and methimazole + T4 (MT4; B: n = 8). Values are shown as mean ± s.e.m. Letters indicate significant differences between treatments; experiments were analyzed separately.

Methimazole treatment effectively suppressed gonadal growth in the inhibition experiment (Fig. 2; t_10_ = −21.09, p < 0.001). In the thyroid replacement experiment we detected significant differences between treatment groups (Fig. 2; F_3,25_ = 159.50, p < 0.001). Methimazole treatment suppressed gonadal growth (diff = −5.16, p < 0.001) as seen in the first experiment. We found significant gonadal growth in both MT4 (diff = 5.71, p < 0.001) and MT3 (diff = 1.34, p = 0.002) birds compared to the Methimazole group. However, only MT4 treatment fully rescued gonadal growth, showing no difference from controls (diff = 0.55, p = 0.32). MT3 treatment did not restore gonadal growth to the level observed in control birds (diff = −3.82, p < 0.001) nor MT4 levels (diff = −4.37, p < 0.001).

### 3.3 *Molt*

**Fig. 3:**
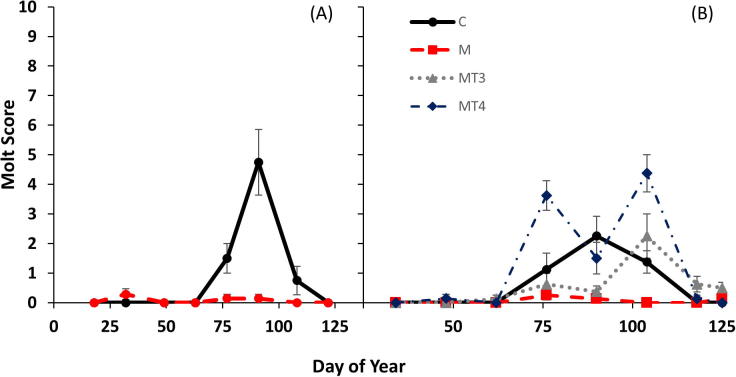
Body molt score for Gambel’s White-crowned sparrows during methimazole inhibition of thyroid hormone (A) and thyroid replacement experiments (B). Treatments include: control (C), Methimazole (M), Methimazole + T3 (MT3) and Methimazole + T4 (MT4). Experiments were analyzed separately. Values displayed as means ± s.e.m.

Inhibition of thyroid hormone production by methimazole (Experiment 1) led to a suppression of pre-nuptial molt over the course of the experiment (Fig. 3A; z = −2.59, p < 0.01). We also detected a significant main effect of day of year (z = 2.11, p = 0.035). In the thyroid replacement experiment (Experiment 2), methimazole treated birds again displayed suppressed molt (Fig. 3B; z = −2.85, p = 0.004) compared to controls. MT3 birds showed no significant difference in peak molt intensity compared to control birds (z = −0.07, p = 0.948), while MT4 birds showed a trend towards increased molt intensity (z = 1.73. p = 0.083). Day of year (z = 1.46, p = 0.14) was not significant nor was the treatment by day of year interaction.

### 3.4 Luteinizing hormone

**Fig. 4:**
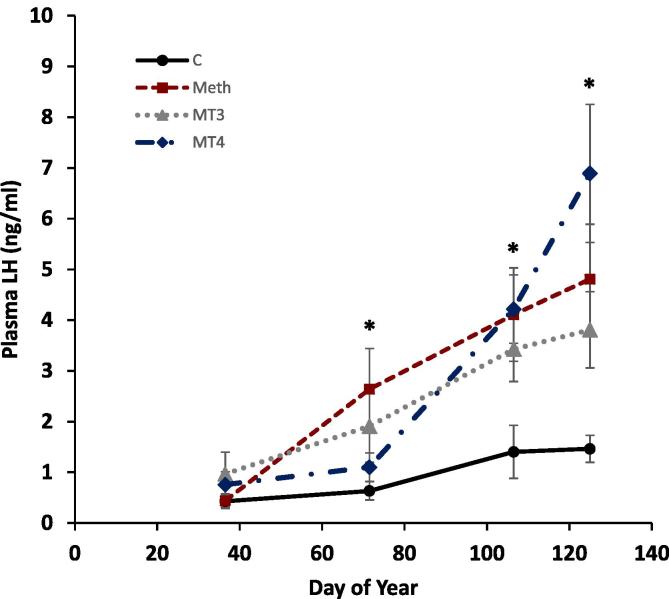
Plasma Luteinizing Hormone (LH) levels by Day of Year for male Gambel’s White-crowned sparrows during Experiment 2: Thyroid Replacement. Treatments included control (C; circles), methimazole (Meth, squares), methimazole + T3 (MT3, triangles) and methimazole + T4 (MT4, diamonds), n = 8 for all groups. Values displayed as means ± sem. Significant group differences from the control group are indicated by “*”.

LH was significantly higher in MT4 birds compared to controls (Fig. 4; t_113_ = −2.45, p = 0.016). The interaction of MT4 and day of year (t_88_ = 5.04, p < 0.001) as well as the methimazole and day of year interaction (t_87_ = 3.10, p = 0.003) were significant, indicating more rapid increases in LH in these groups compared to controls. The interaction of MT3 treatment and day of year was not significant (t_88_ = 1.94, p = 0.056), but showed a trend towards significance. There was no significant difference between treatment groups at the beginning of the experiment (F_3,25_ = 1.06, p = 0.383). LH levels in the treatment groups became significantly different at the day of year 106.5 (F_3,28_ = 4.562, p = 0.01) and persisted until the end of the experiment (Day of year 125; F_3,26_ = 6.95, p = 0.001).

## 4 Discussion

Methimazole inhibition of TH production prevented both pre-nuptial molt and gonadal recrudescence. These results are consistent with previous findings demonstrating a central role for hypothalamic thyroid signaling in controlling the HPG axis (Nakane and Yoshimura, 2010, Perfito et al., 2014, Wilson and Reinert, 1995, Wilson and Reinert, 1996). These findings also demonstrate that TH (T3 or T4) are necessary for the expression of pre-nuptial molt in white-crowned sparrows. This observation is consistent with existing knowledge from feather replacement experiments and investigations of post-nuptial molt, while extending the necessity of TH action for pre-nuptial molt as well.

Contrary to our predictions only T4 administration restored gonadal growth. Previous work has shown that ICV administration of T3 into the third ventricle of American tree sparrows (*Spizella arborea*) triggers a robust LH surge and gonadal growth (Wilson and Reinert, 2000). This discrepancy between the effects of ICV and peripheral administration of T3 suggests an inability of T3 to cross the blood brain barrier. Conversely, the efficacy of T4 replacement treatment in restoring gonadal growth in chemically thyroidectomized birds supports the notion of T4 entry into the hypothalamus where it is presumably converted to T3 for local signaling (Yasuo et al., 2005, Yoshimura et al., 2003). The observed differential action of T4 and T3 supports direct control of TH access to the brain presumably via membrane transporters in the blood brain barrier. The presence of TH membrane transporters, along with the thyroid binding protein transthyretin, has been demonstrated in the choroid plexus of chicks (Duan et al., 1991, Power et al., 2000) and in the capillaries of the blood brain barrier itself (Van Herck et al., 2015), further supporting this hypothesis. However, the identity, presence, and affinity for T4 versus T3 of TH membrane transporters has yet to be confirmed in adult birds. Studies of known membrane transporters including OATP2B1 and OATP3_v1/v2 have demonstrated variable affinities for a number of iodothyronine derivatives, in particular specificity for T4 over other thyroid derivatives (reviewed in Visser et al., 2011). In mammalian models MCT8 is considered the primary membrane transporter and has demonstrated a higher affinity for T4 (Müller and Heuer, 2014). Selective transport of T4 into the hypothalamic region would simultaneously explain our findings and the observation that direct infusion of T3 is able to induce a HPG response akin to photostimulation, while peripheral administration of T3 fails to do so. This is further supported by our finding that both T3 and T4 restore normal molt, which eliminates the possibility of exogenous T3 being cleared by the liver prior to reaching target tissues. Such a selective control mechanism of TH transport into the hypothalamus, if not the whole brain, is heuristically appealing, as it would prevent the continuous activation of the HPG axis and other systems in response to T3 circulating at normal levels. Instead, under such a model local deiodinase activity would determine which brain regions experience TH signaling.

Though confounding and initially inconsistent with the above interpretation of our gonadal data, the observed LH values must be viewed with caution. The lack of a definitive peak of LH in control birds appears initially inconsistent with the numerous studies that report a rapid rise in LH within 18 h of photostimulation (e.g. Follett et al., 1974). However, these previous studies used immediate shifts in photoperiod, transferring birds to sustained day lengths far longer than would be experienced under natural conditions directly from short days, all within the span of a single day, while the present study utilized natural photoperiod, making direct comparisons questionable. Evidence to support this view was published by Wingfield and Farner (1978) in which LH levels of Gambel’s white-crowned sparrows measured in the field did not peak until the period of territory establishment and egg laying. Given our use of a naturally increasing photoperiod, the low LH levels might then be expected, as the experimental period did not extend past the early migratory period (May 5th) and wild populations do not arrive on their breeding grounds till late in May.

The significant increase in circulating LH in all three treatment groups was also unexpected. However, the observed LH elevation following methimazole treatment may be explained by cross reactivity of TSH with the LH antibody employed in our radioimmunoassay. While such cross reactivity has not proven problematic in past reports of this assay, initial validations demonstrated detectable cross reactivity with TSH at high levels, as might be expected during thyroidectomy (Sharp et al., 1987). Chemical inhibition of TH by methimazole inherently suppresses the normal negative feedback of TH on the hypothalamic-pituitary-thyroid axis, resulting in elevation of TSH in an attempt to restore homeostatic balance. Thus high TSH levels might explain the observed LH increase in the methimazole treated birds. Similarly the observed increase in either MT3 or MT4 birds may also be due to suppression of negative feedback on TSH. If membrane transporters selectively control exposure of thyrotroph cells to TH then we might expect birds given the non-transported form of TH to experience an increase in TSH and thus observed LH similar to methimazole treated birds. However, this hypothesis still fails to explain the observed increase in the remaining replacement group (either MT3 or MT4).

Alternatively there may be direct interference by methimazole. Though TSH interference was suggested by Sharp et al. (1987), avian TSH had not been purified at the time, thus in demonstrating the supposed cross-reactivity of TSH with the LH assay, they relied on methimazole to trigger increased TSH by inducing hypothyroidism. This raises the possibility that TSH effect Sharp et al. (1987) observed was at least in part direct interference by methimazole. However, this is countered by the observation that methimazole treatment is able to suppress gonadal growth and LH in both rats and Japanese quail, when measured using alternate LH assays (Valle et al., 1985, Weng et al., 2007). Furthermore, Valle et al. (1985) observed that methimazole treatment directly impacted LH receptor density within the testis of immature rats, and that LH receptor density remained decreased even in response to replacement treatment with T3. However, the Leydig cells from these animals produced testosterone normally in response to stimulation with a hCG antagonist in culture. Thus the possibility of direct action by methimazole on the testes through LH receptor density or other pathways cannot be ruled out. However, the differential response to T3 and T4 replacement therapies supports alternative explanations. While the exact cause remains unclear, it appears that chemical thyroidectomy with methimazole has pleiotropic effects across a number of systems and tissues that complicates interpretation of simple physiological and hormonal responses to its use. Given the complex interrelationships of HPT activity and gonadal function, the LH data remain unexplained. Therefore, future work is urgently needed to disentangle the effects of systemic TH suppression by methimazole on LH and more broadly on the HPG axis. This work may be particularly fruitful in revealing novel mechanisms of HPG and HPT cross talk and regulation.

Lesioning studies of the ventral medial hypothalamus (VMH) in white-throated sparrows have identified this as a site of central control of pre-nuptial molt (Kuenzel, 1974). However, our data suggest that TH control of pre-nuptial molt is peripheral, likely at the level of the feather follicle, rather than by central action of TH, because of the non-differential response of MT3 and MT4 treatments. This view is further supported by our observation of differential response to T3 and T4 replacement treatments for testicular growth. If the proposed mechanism of variable transport of T4 versus T3 across the blood brain barrier explains our observation of the inhibition of testicular growth results, then the observation of equivalent rescue by both T3 and T4 replacement for pre-nuptial molt is most parsimoniously explained by peripheral rather than central action.

## 5 Conclusions

Together these results further support the involvement of TH in the regulation and control of both the breeding and pre-nuptial molting life history stages. The conflicting effects of T3 versus T4 replacement with regards to testicular growth and LH levels highlight the importance of multi-level regulation of the thyroid system. Furthermore, THs are likely acting at the organizational level during initial photostimulation influencing the development and full expressions of molt and gonadal recrudescence and later to trigger the onset of photorefractoriness (Dawson et al., 1985, Follett and Nicholls, 1985, Follett and Nicholls, 1988) and future work should seek to disentangle these two processes. Furthermore, these results emphasize the complexity of the HPG and HPT axes, their regulation and interplay that urgently require further study. A detailed understanding of transporter, deiodinase, and receptor regulations spatially and temporally within target tissues will be critical to further explanations of the unique role of thyroid hormones in regulation of seasonal transitions.
